# Open Problems in Computational Historical Linguistics

**DOI:** 10.12688/openreseurope.16804.2

**Published:** 2024-05-29

**Authors:** Johann-Mattis List

**Affiliations:** 1 Chair of Multilingual Computational Linguistics, University of Passau, Passau, Bavaria, 94032, Germany; 2 Department of Linguistic and Cultural Evolution, Max Planck Institute for Evolutionary Anthropology, Leipzig, 04103, Germany

**Keywords:** historical linguistics, computational linguistics, open problems, scientific problem solving

## Abstract

Problems constitute the starting point of all scientific research. The essay reflects on the different kinds of problems that scientists address in their research and discusses a list of 10 problems for the field of computational historical linguistics, that was proposed throughout 2019 in a series of blog posts (see http://phylonetworks.blogspot.com/). In contrast to problems identified in different contexts, these problems were considered to be solvable, but no solution could be proposed back then. By discussing the problems in the light of developments that have been made in the field during the past five years, a modified list is proposed that takes new insights into account but also finds that the majority of the problems has not yet been solved.

## Introduction

The driving force of many scientific inquiries are problems – unsolved or *open problems*. We observe phenomena that we cannot explain, we do not understand how phenomena interact and to which degree they influence each other, or we want to know how we can enhance methods that allow us to study phenomena of interest. While questions that ask for the solution of big problems often trigger peoples’ initial interest in science, scientists themselves typically work with much smaller problems. While the solution of small problems may seem boring to the public, they are crucial for the field to advance and they can lay the foundation for major breakthroughs at later stages.

Working on the typically small problems that individual subfields offer, all scientists run the risk of loosing track of their discipline’s broader challenges. Working fortunately in our ivory tower that shields us from the outside world, it is often through confrontation with laypeople or scientists from other fields that we are made aware of the greater challenges of our own discipline.

In the field of linguistics, specifically in the field of historical linguistics, one of the questions that linguists have stopped asking is how language – that is, the specific communicative faculty of which many think is unique to humans – originated for the first time. Non-linguists are often very surprised that asking for the origin of language has been a taboo question in the field of historical linguistics for a very long time. Already in the 19th century, the question had been officially banned from the agenda of most linguistic endeavors. The *statuts* of the *Société de Linguistique de Paris* from 1866, for example, state that ‘[the] Société does not accept any contributions either on the origin of the language faculty, or the creation of a universal language.’ ( Société de Linguistique de Paris, 1871, III). ^1^ In my impression, this situation has not changed significantly since then. While — as reviewers of the first version of this essay have pointed out — it is true that the interest of scientists who discuss how language originated the first time has increased (which is also reflected in journals dedicated to the topic and in conferences devoted to it), the discipline of historical linguistics in particular and mainstream linguistics in general still largely avoid the question, reflecting the Société's ban until today. One of the first things I learned as a student of Indo-European and Comparative Linguistics at the Freie Universität Berlin in 2003 was that serious historical linguists would never ask about the origin or evolution of language. Since then, I have often experienced dismissive reactions, specifically in the German university context, when talking about *language evolution* instead of *language history*. Major textbooks in historical linguistics exclude the question of how language originated completely, and some even mention this explicitly, as reviewer Michael Pleyer has pointed out ( Campbell, 1999[1998]: 1f)

Of course, there are good reasons to avoid asking how language originated. When sifting through the literature that has been published on this matter, one finds a diverse collection ranging from serious attempts to summarize what we can know and what we can’t know, up to the weirdest speculations. There are not many similar fields in science where it is so difficult to draw a line between genius and madness. On the one hand, one finds researchers who meticulously assemble tiniest pieces of evidence in the search for a clear picture in the dark glasses. On the other hand, one finds scholars so obsessed with a single idea that they have become blind to counterarguments. Specifically for outsiders from the field, for those not trained in historical linguistics and evolutionary anthropology, it is often very difficult to tell if a theory on the origin of language should be treated as a serious or a senseless idea. Next to Johann Gottfried Herder (1744–1802) who imagined that human beings would have run through the woods imitating the sounds of the objects and phenomena surrounding them ( Herder, 1778, see also Table 1), scholars discuss whether Neanderthals could speak or not ( Dediu & Levinson, 2013), they have debated for some time whether a single gene was responsible for our language faculty ( Atkinson *et al.*, 2018), they propose that research on aphasia might shed light on the structure of early language ( Code, 2021), and they speculate about the nature of the ‘language of proto-sapiens’ in the context of *yin* and *yang* ( Papakitsos & Kenanidis, 2018, see also Table 1). There is no doubt: the question invites bold speculation, and when bold speculation becomes careless, it can easily damage the reputation of entire scientific fields. For outsiders who do not know the scientific community that tries to investigate the question of the origin of language carefully, it is often not easy to distinguish between serious and highly speculative attempts. At the same time, however, it feels strange that linguists would deliberately decide to ignore the investigation of a problem that some might consider the most fascinating the field has to offer. While following inconclusive debates and trying to not get angry or amused by weird speculations regarding the “big questions” of one’s discipline, one may easily forget that there *are* valid and important problems which one may have forgotten to think about. An example for such a problem that is routinely ignored in historical linguistics is the problem of the “size” of a language. While historical linguists hardly ever ask how many words a language has, or how many sentences one can speak until one would repeat oneself, non-linguists have often asked me these questions. I still remember quite vividly how annoyed I always felt – and at times even reacted – when colleagues from biology asked me if English was bigger than German or French. I tried to explain that languages are open systems that cannot be measured by counting the words in a corpus and that if at all, one would have to count the words in the head of individuals, but that this would not be possible from a practical viewpoint, and that as a result of these complications, linguists preferred to ignore the question completely and look into other problems instead. But with time, I learned to put my linguistic pride aside and began to understand that a solution to the question would have several important implications to other questions that are of vital interest to my research.

**Table 1.** Collection of quotes on the origin of language.

Sound imitation at the origin of language.
*Take for example the sheep. As an image [...] – how much, how difficult to discern! All characteristics are intertwined, next to each other, all* *still unspeakable! Who can talk shape? Who can sound colors? [...] Who can say what he feels with his hands? But listen, the sheep bleats! [...] "Ha!" says the apprentice [...], "now I will recognize you – you bleat!" The turtledove coos! The dog barks! There are three words, because he was* *looking for three clear ideas, the latter go into his logic, the former into his dictionary! [...] The soul grasped [for it] – there it has a sounding* *word!* ( Herder, 1778, 76). ^2^
Yin, yang, and the language of proto-sapiens.
*The Proto-Sapiens grammar was so simple that the sporadic references in previous paragraphs have essentially described it. The prime* *importance of sound symbolism for the people of nature should be noted again before we further detail that the vowel E was felt as indicating the “yin” element, passivity, femininity etc., while “O” indicated the yang element, activeness, masculinity etc.; “A” was neutral or spiritual,* *indicating things conceived by the mind and emotions rather than with the physical senses.* ( Papakitsos & Kenanidis, 2018, 8)

In evolutionary biology, for example, scholars have argued that the number of genes that were horizontally transferred among species largely exceeds the number of genes that were vertically inherited ( Dagan & Martin, 2006). Horizontal transfer is quite abundant in language history as well, and we usually base our phylogenetic studies on very small collections of basic words often not even exceeding 200 items per language ( Greenhill *et al.*, 2023; Sagart *et al.*, 2019). It would therefore be interesting to have a rough estimate of how many words of a language survive over time, but this would require (at the very least) a rough estimate of the words that constitute a language.

In historical linguistics, Starostin (1989) has proposed that every language has about 1000 lexical roots from which most of the words in the language are formed. Up to today, no attempt has been made to count the number of lexical roots, even for well-documented languages like German or Chinese. It would be very interesting to see if Starostin’s estimate holds cross-linguistically, and how much variation we should expect when comparing the languages of the world.

Given that languages may differ quite substantially regarding the way in which they build new words from existing ones, it would also be very interesting to see to which degree languages differ regarding the “productivity” of their words to form families ( List, 2023), and to which degree the production of new vocabulary is triggered by external events, such as, for example, pandemics or wars.

Lastly, measuring the number of words that different speakers of the same language know might even help us to investigate to which degree individual language faculties vary among humans. The question to which degree speakers of the same language differ regarding their competence is another question that is – unfortunately – rarely asked, although the notion of competence plays a crucial role in some linguistic fields. ^3^

While the question of the “size of a language” has been mostly ignored in the context of historical linguistics (Deutscher's assessment that literacy plays a major role in vocabulary size is a rare exception that does, however, not attempt to demonstrate the claim empirically, see Deutscher, 2010: 111), vocabulary size or vocubulary breadth ^4^ have been routinely investigated by scholars focusing on foreign language acquisition ( Milton, 2009) and psycholinguistics ( Brysbaert *et al.*, 2016; see specifically also Nation & Coxhead, 2021 on the problems of estimating vocabulary sizes). Unfortunately, the majority of these studies has concentrated exclusively on English. We know that languages may differ quite substantially regarding the structure of their word families and the techniques they use to create new words from existing ones ( Milton, 2010, 226, see also List *et al.*, 2016b, 9f). As a result, the estimates on vocabulary size are of limited use to address the above-mentioned problems in historical linguistics, and it remains an open problem to measure and compare the size of the vocabularies of the worlds’ languages.

As scientists, we cannot ask enough questions. Being used to address small problems as part of our scientific routines, however, we may forget to ask the “big questions” that are at the heart of our particular disciplines. By ignoring certain questions deliberately and limiting the scope of questions we allow ourselves to ask in our work we may easily lose the chance to enrich our studies and open new horizons. Specifically for young and rapidly growing subdisciplines such as the field of computational historical linguistics, it can be very helpful to identify particular problems and challenges that should be addressed in future work. When talking about computational historical linguistics in this context, I refer to those attempts that try to formalize and automate the classical approaches for historical language comparison that have been developed in the traditional historical linguistics (often referred to as the "comparative method"). In the following, I will reflect on challenges that have been discussed in the context of comparative linguistics an contrast them with those challenges that I have identified for my own work. In doing so, I hope to show that an active discussion about open problems can be a useful guiding principle not only for an entire research field, but also for individual researchers.

## Rational, general and historical problems

Major problems and challenges for the field of comparative linguistics have been discussed in the past on different occasions. Weinreich *et al.* (1968, 183–187) identify five general problems with respect to the phenomenon of language change, which they call

(1) the *constraints problem*, dealing with the question of which changes are possible in language change and which conditions could constrain them,
(2) the *transition problem*, dealing with the question of how and where changes are instantiated in concrete during language change,
(3) the *embedding problem*, dealing with the systemic aspects conditioning language change,
(4) the *evaluation problem*, dealing with the question to which degree communities of language users are aware of the changes that are happening, and
(5) the *actuation problem*, dealing with the question of how changes are triggered.

Roberts and Sneller (2020) discuss these in the context of the “four questions” for evolutionary sciences proposed by Tinbergen (1963), pointing to potential empirical implications and programs for future research. Tinbergen’s questions themselves have been originally stated for the field of biological evolution ( Bateson & Laland, 2013), although they were later adopted by researchers studying cultural evolution ( Roberts & Sneller, 2020, 194f). They are nowadays usually presented in a more systematic fashion than the problems by Weinreich *et al.* (1968), distinguishing two major perspectives, *dynamic* (diachronic) and *static* (synchronic), and two major kinds of questions, *how-* and *why-*questions, the former referring to individuals and the latter to species ( Tinbergen, 1963). This allows to look at particular problems (e.g., the evolution of a specific trait) from four perspectives, namely

(1) the perspective of the *ontogeny*, focusing on how the trait evolves in individuals,
(2) the perspective of the *mechanism*, focusing on how the trait is structured synchronically in an individual,
(3) the perspective of the *phylogeny*, focusing on how the trait evolves inside a given species, and
(4) the perspective of the *function*, focusing on the role adaptive role the trait plays for a given species.

Similar to Roberts and Sneller (2020), I do not find it very helpful to try to classify the five problems by Weinreich *et al.* (1968) according to the schema proposed by Tinbergen (1963). Despite the apparent systematicity of the latter, I find the schema hard to apply to concrete problems.

As yet another example for an attempt to systematize linguistic endeavor by stating problems, Eugenio Coseriu (1921–2002, see Coseriu, 1973, 65f) suggested distinguishing three basic problems of language change, namely

(a) the rational problem of change (“problema racional del cambio”),
(b) the general problem of change events (“problema general de los cambios”), and
(c) the historical problem of a given change (“problema histórico de tal cambio determinado”).

These problems can again be represented by certain questions, as indicated by Coseriu himself. The rational problem asks why languages change after all (“¿por que cambian las lenguas?”). This question does not find a counterpart in the list of problems proposed by Weinreich *et al.* (1968), where language change has been taken for granted, and the goal is to investigate and describe the phenomenon. As Coseriu emphasizes himself, the problem is of a chiefly *theoretical* nature and cannot be resolved by identifying all causes for particular changes that can be observed for particular languages (ibid. 66f), but rather addresses the deeper question of why mutability is one of the fundamental characteristics of language (68f). Coseriu himself sees the reason for the mutable character of language as a result of the fact that languages is constantly recreated, not only when being learned by speakers, but also when being applied by them (69f).

The second problem of Coseriu is similar to the *actuation problem* by Weinreich *et al.* (1968), addressing the question in which conditions certain changes occur (“¿en qué condiciones suelen occurrir cambios en las lenguas?”). In Coseriu’s view, this problem is a problem of *general linguistics* in the sense that general linguistics deals with linguistic phenomena independently of particular languages. Particular changes in particular languages, finally, are addressed by the third problem, which Coseriu calls *historical*, emphasizing the individual character of investigating particular changes in particular languages.

Coseriu’s strict distinction between *general problems* in linguistics on the one hand and *historical problems* on the other hand finds a very close counterpart in the distinction between *p(articular language)-linguistics* and *g(eneral) linguistics* by Haspelmath (2020, 5), where a distinction between the investigation of language as a general communication system and the investigation of individual languages is made (see also Haspelmath, 2019).

Judging from numerous discussions with colleagues, distinguishing questions applying to individual languages and language families from questions applying to language in general (as a system of human communication) constitutes a much more important systematization of problems than the attempts by Weinreich *et al.* and Tinbergen. The failure to distinguish questions pertaining to particular languages and questions pertaining to language in general has led to many misunderstandings in the field of comparative linguistics. In my own research, it has happened quite a few times that scholars who reviewed my work were asking me to test new methods which I had designed to account for general problems against previously proposed methods that could only solve problems for particular languages (at times even relying on particular orthographies, while my methods would use phonetic transcriptions). It also happens a lot — specifically when presenting new methods to computational linguists who do not know the particular problems of historical linguistics very well — that newly proposed general methods that work in an unsupervised fashion (i.e., without requiring training data) are rejected with the justification that supervised methods that solve the problem for particular languages (using a substantial amount of training examples) exists.

## Hilbert problems

At the end of 2018, students from the Universidad de Buenos Aires asked me about the biggest challenges for computational historical linguistics. Inspired by this discussion, I decided to make a short list of tasks that I consider challenging, but of which I still think could be solved some time in the nearer or farther future.

The idea to make such a list of questions is not new. Mathematicians, for example, have their well-known *Hilbert Problems*, proposed by David Hilbert in 1900 (published in Hilbert, 1902). In linguistics, I first heard about them from Russell D. Gray, who himself was introduced to this by a talk of the linguist Martin Hilpert, who gave a talk on challenging questions for linguistics in 2014, called “Challenges for 21st century linguistics". Russell D. Gray since then has emphasized the importance to propose “Hilbert" questions for the fields of comparative linguistics and cultural evolution, and has also presented his own challenges in the past.

Due to my methodological background, the problems I identified and assembled were by no means big and in some sense also not necessarily extremely challenging (at least not at first sight). Instead, the problems I selected were problems I wanted to see solved at that time. While the solution of the problems would not directly advance our knowledge about language evolution and linguistic typology, I had the hope that it would help us to do so indirectly, by giving us the possibility to assemble more data and to carry out new analyses that would ultimately help us to search for answers on deeper questions in historical linguistics in specific and in comparative linguistics in general. Due to this goal of providing solutions for historical linguistics in general, I was interested in solutions that could applied to any language, as long as it is represented in some uniform way (in phonetic transcription rather than orthography). Open problems also exist in the context of particular languages, but my interest was in problems in general historical linguistics rather than historical linguistics of particular language families.

One further aspect of the problems that I selected was that I was convinced that they could *all* be solved by algorithms or workflows. Characterizing them as “small” refers to their very specific application range. I did not want to express that the problems I selected were not challenging. It also did not mean that I expected that they all could be solved in the nearer future, although, given that the work in the field of computational and computer-assisted language comparison, is steadily progressing, I had some confidence that at least some of these problems that I assembled by then would indeed be solvable within the next five years.

When writing down my ten open problems for computational historical linguistics, I announced them in a blog post for the blog *The Genealogical World of Phylogenetic Networks* ( https://phylonetworks.blogspot.com/), edited by David Morrison, in January 2019 ( List, 2019a), with the plan of discussing each of the problems in detail in monthly blog posts throughout the year. I managed to stick with this schedule and concluded the year with a final blog post in December 2019, in which I looked back at one year of discussing problems in my own research for which no solution could have been found by then ( List, 2019d).

**Figure 1.**
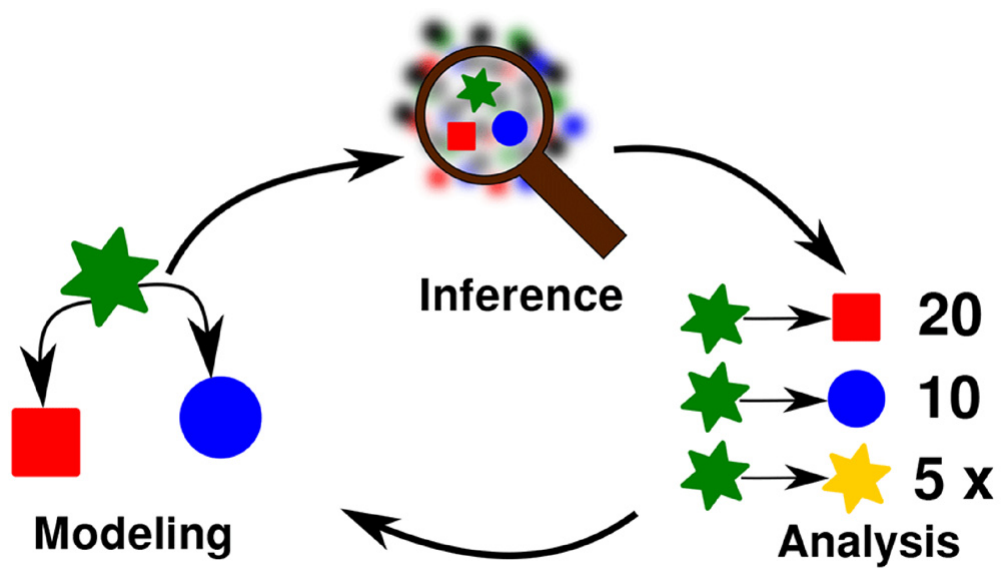
Research workflow for the investigation of problems in computational historical linguistics. The stage of modeling assumes certain relations between certain scientific objects. The stage of inference tries to find more examples for the relations proposed by a given model. The stage of analysis would then — for example — compare the frequency by which different processes can be accounted for and use this information to inform the model.

The 10 problems I came up with are listed in Table 2. I divided the problems into three different groups, which roughly correspond to three different categories I identified as being important for research in general, namely *modeling* (m), *inference* (i), and *analysis* (a). This triad, inspired by Dehmer *et al.* (2011, XVII), follows the general idea that scientific research in the historical disciplines usually starts from some kind of idea we have about our research object (the *model* stage), and based on which we then apply methods to infer examples in our data which confirm our ideas (the *inference* stage). Having inferred enough examples, we can then *analyze* them qualitatively or quantitatively (the *analysis* stage) and use this information to update our model, as indicated in the schema in Figure 1.

**Table 2.** 10 problems of computational diversity linguistics discussed in a series of blog posts in 2019. Problems labeled as (i) are inference problems, problems labeled (m) refer to modeling problems, and problems labeled (a) are analysis problems.

No.	Problem
1	automated morpheme segmentation (i)
2	automated borrowing detection (i)
3	automated sound law induction (i)
4	automated phonological reconstruction (i)
5	simulating lexical change (m)
6	simulating sound change (m)
7	statistical proof of language relatedness (m)
8	typology of semantic change (a)
9	typology of sound change (a)
10	typology of semantic promiscuity (a)

As an example for this procedure, consider the problem of *cognate detection*, the detection of historically related – or homologous – words across languages. Here, initial surveys of words across different languages have long since confirmed that we can easily find words which are to some extent similar to each other with respect to their form and their meaning, and that the amount of similar words varies quite significantly from one pair of languages to another. As an example, consider word pairs, such as German *Zeh* "toe" and English *toe*, German *zwei* "two" and English *two*, or German Zeichen "sign" and English *token*. Starting from a model of *lexical change* that states that the lexicon of all spoken languages changes slowly over time, be it through the change of individual sounds or through the change of the meaning which a word expresses, we can derive a model of *language split* which assumes that the same language may split into two or more varieties when its speakers separate from each other and their language keeps modifying independently. Based on this model, we can then conclude that similar words observed across different language varieties have been inherited from the common, formerly unified, ancestral variety. In order to detect these *cognates*, one could now design methods that help to find more than the so far observed similar words in order to increase the data basis. In the case of German and English, one could design a method by which dictionaries in English and German are searched for words that start with *z-* in German and with *t-* in English and compare their semantics. Once this has been done, and more material has been identified, one can *analyze* the data and try to see if the analysis provides some hints on specific questions, such as, for example, the more detailed branching history of a given language family, or to which degree language contact has masked further evidence. More examples of German words containing a *z* with counterparts containing a *t* in English would, for example, show that there are more nuances to the correspondences (compare cases like German *heiß* "hot" vs. English *hot*, or German *hassen "hate" vs. English "hate"*), and that semantics can vary considerably (compare German *Zaun* "fence" vs. English *town*).

As one can see from the example, the term *modeling* is used in a rather loose sense, unlike its usage in phylogenetic approaches, where *modeling* is often treated as synonymous with *stochastic modeling*, referring to a transition matrix which shows how certain characters can turn into other characters during an evolutionary process ( Nunn, 2011).

With respect to my ten open problems from 2019, the first four problems belong to the family of inference problems, since they all deal with tasks where something has to be inferred from the data, be it morphemes from words (Problem 1, List, 2019e), lexical borrowings from word lists (Problem 2, List, 2019g), sound laws from data on ancestral languages and their descendants (Problem 3, List, 2019f), or proto-forms from cognate sets (Problem 4, List, 2019h). Note that the inference of morphemes in wordlists is listed as a problem pertaining to the field of historical linguistics here, because the identification of the morphemes in a language is an important step for *internal reconstruction*. Although the handling of morphemes and the identification of the principles underlying word formation in particular languages is often seen as a classical problem in synchronic linguistics, such analyses are typically diachronic in nature, or — to put it in other terms — it is not always easy to distinguish diachrony from synchrony when dealing with questions of morphology and word formation. Note also that not all linguists agree on the notion of the morpheme. From the perspective of historical linguistics, where one must try to control for allomorphic alternation, the notion of morpheme boundaries and morpheme segmentation is crucial, even if it is rarely discussed in classical handbooks introducing the traditional methodology of historical language comparison.

The next three problems in my list belong to the family of modeling problems, since they all require to understand the processes by which certain aspects of languages, such as their lexicon (Problem 5, List, 2019i) or their sound systems (Problem 6, List, 2019j) change over time. Proving language relatedness (i.e. that two or more languages have descended from the same ancestral language) statistically (Problem 7, List, 2019k) does not directly model any aspect of language evolution, but it requires a model of language relatedness that can then be tested against a random model in which languages are thought to be unrelated. ^5^

The last three problems in my list all had “typology” in their title. They belong to the family of analysis problems, aiming to gain insights into phenomena of language change by comparing major processes, such as semantic change (Problem 8, List, 2019l) and sound change (Problem 9, List, 2019b). What was meant by “typology” in this context was a data-driven estimate of the overall cross-linguistic dynamics of these phenomena. Lacking consistent accounts on the general tendencies of these processes and phenomena when excluding areal and genetic factors, the task I thought of ways to come up with a consistent estimate on each of them. While semantic change and sound change are probably self-explaining in this context, the last problem – dealing with the question of what I called “semantic promiscuity” by then – deserves some explanation (Problem 10, List, 2019c). What I meant with this term was the degree to which certain words, due to their original meanings, are re-used or re-cycled in the human lexicon. While the term *promiscuity* has been used before in other contexts in linguistics ( Schweikhard, 2018), the specific usage of promiscuity to denote what one could also call *semantic productivity* or *concept productivity*, was first proposed in List *et al.* (2016a), where biological and linguistic processes were consistently compared with each other, and semantic promiscuity was identified as a phenomenon similar to *domain promiscuity* in protein evolution in biology ( Basu *et al.*, 2008), with an explicit analogy being identified between the processes of *word formation* in linguistics and *protein assembly* ( Ahnert *et al.*, 2015) in biology ( List *et al.*, 2016a, 5). As I'll discuss in more detail below, I now tend to call the phenomenon *lexical root productivity*, a term supposed to denote the propensity of certain words and morphemes to be used to form new words due to the meaning they express.

## New and old open problems

The series of blog posts was generally well received, and some posts triggered interesting discussions. However, my hope that at least a few of the problems might be considered as “solved” after half a decade turned out to have been a false one. When looking back at the list of ten problems now, almost five years after I first proposed them, I do not have the feeling that we are any closer in solving any of them. For this reason, the list of open problems that I consider important for my own field and my own work has remained almost unchanged, although more than five years have passed by now.

## Inference problems

As far as inference problems are concerned, no significant progress was made with respect to the tasks of automated morpheme segmentation (Problem 1) and automated sound law induction (Problem 3). Research on natural language processing has profited from new segmentation approaches that avoid to take the word as the basic unit of texts, proposing to segment texts in large corpora into units beyond the word. These methods, however, cannot be applied to the specific problem of morpheme segmentation outlined in my list of problems, where much fewer words in phonetic transcription constitute the basic unit of analysis.

Quite a few new methods have been proposed to address the detection of lexical borrowings (Problem 2). Among these are supervised approaches (which I had deliberately excluded, since I consider unsupervised approaches as more useful when it comes to inference problems) that made use of recurrent neural networks ( Miller *et al.*, 2020), there are tree-based approaches that even try to identify the direction of borrowings ( Neureiter *et al.*, 2022), ^6^ and there are numerous attempts to handle very specific cases of lexical borrowing, such as contact across language families ( Hantgan *et al.*, 2022; List & Forkel, 2022) or contact induced by dominant languages ( Kaiping & Klamer, 2022; Miller & List, 2023).

While all of these methods may contribute to the detection of borrowings in particular cases, all of them suffer from the problem that they need very specific conditions to work. For supervised approaches, we need labeled training data, for tree-based approaches to borrowing detection, we need phylogenetic trees, for borrowing detection across language families, we need languages to belong to different families, and for the detection of borrowing from dominant languages, we need to deal with languages that are spoken in a region where dominant languages occur.

It is well possible, that the current pocket knife solution that employs various forms of evidence to find borrowings in very specific contexts is the only feasible way to handle the problem of language contact in computational historical linguistics. Even classical approaches to historical language comparison do not use one unified approach to identify borrowed traits, but rather hope to accumulate enough evidence until borrowing is the only convincing solution to explain the data at hand. But even if we accept that we need to embrace arguments based on “cumulative evidence” ( Berg, 1998) or “consilience” ( Whewell, 1847; Wilson, 1998) in order to solve the problem of borrowing detection (see also List, 2019n, 11), we are still quite far away from being able to handle all the evidence with automated approaches which goes into the argumentation of classical qualitative approaches to borrowing detection. From today’s perspective, I would adjust the problem of borrowing detection in order to make it more specific. Here, a problem I would really love to be solved would be the detection of the *layers of contact* across a group of languages ( Lee & Sagart, 2008). Contact layers have been discussed for a long time in the literature on language contact. The idea behind contact layers is that the individual traits of a language can be *stratified* and assigned to different groups that would point to different phases in which these traits were borrowed through specific contact events. Developing a method that would be able to group borrowings into different strata that could then be identified with specific contact events in time would be extremely beneficial for the discipline of historical linguistics. The problem is, however, also very challenging, since it is not clear whether contact layers *can* be identified in all cases (evidence might just have been lost), and on what kind of evidence one should base the detection of contact layers. This makes contact layer detection a truly hard problem, although I would not consider it impossible to solve.

Rather huge progress – at least when looking back specifically at the last couple of years – has been made with respect to supervised phonological reconstruction. Here the task is different from the problem I had originally stated. Instead of inferring the proto-language from a sufficiently large number of aligned cognate sets, the method is given aligned cognate sets (or simply cognate sets) along with a certain number of already reconstructed proto-forms that can be used to train a machine learning model in a first instance. In a second instance the model can then be used to infer proto-forms for data that has not been seen previously.

While scholars had been working on this problem before, a first impressive demonstration of the capability of machine learning methods was done by Meloni *et al.* (2021) for a test set in which Latin words needed to be reconstructed from words in several Romance languages. The authors used recurrent neural networks to learn to predict Latin word forms from word forms in the Romance languages. What was remarkable about their approach was the high accuracy reported. Using the edit distance ( Levenshtein, 1966) to compare the proposed Latin word with the attested Latin word, their best parameter settings reported scores in which the proposed word would on average diverge less than one character from the attested word form. Kim *et al.* (2023) repeated the experiments by Meloni *et al.* (2021) but used Transformers ( Vaswani *et al.*, 2017), the famous architecture for neural networks, that has revolutionized artificial intelligence applications in many areas, but is best known in the context of large stochastic language models that are used to run chat bots. Not unexpectedly, the Transformer models further outperform the recurrent neural network architecture employed by Meloni *et al.* (2021).

The problem of supervised phonological reconstruction can be stated in the broader context of *reflex prediction*. Reflex prediction refers to the task of predicting how a word sounds when knowing the pronunciation of historically related words in related languages. For example, observing words like German *Zoll* and Swedish *tull* “customs”, I might predict that the English should have a corresponding words *toll* with a meaning similar (but not necessarily identical) to the meaning of the words in Swedish and German. At times, this may even work with language pairs, and as learners of languages closely related to languages we know intimately, we may even intuitively predict how certain words in the foreign language might sound, based on our knowledge of similar words in the languages we know.

We introduced the task in a preregistered study in which we first predicted word forms in languages that had so far been insufficiently studied and then checked the predictions against word forms verified in field work carried out after the predictions had been registered ( Bodt & List, 2022). For the prediction, we used a supervised approach that goes back to a new method for the detection of regular sound correspondence patterns which I had introduced in 2019m. Having refined this approach in a later study, testing it on a larger collection of datasets from different language families ( List *et al.*, 2022b), we used it as a baseline for a *shared task on reflex prediction* where we invited scholars with a background in machine learning and historical linguistics to design their own approaches for the task of reflex prediction ( List *et al.*, 2022c). For this shared task, we used an even larger number of datasets from the Lexibank repository ( List *et al.*, 2022a) and introduced a computer-assisted pipeline to make sure that all participants would have access to the data in the same form. The best systems were presented by Kirov *et al.* (2022) who used sophisticated neural network architectures and data processing pipelines to augment the very sparse input data. Interestingly, their best model was not based on transformers, but on convolutional network architectures originally designed for the task of restoring images ( Liu *et al.*, 2018). While the recent success of neural network approaches in the task of phonological reconstruction and reflex prediction is definitely impressive, the approaches have the drawback of not telling us how they arrive at their decisions. The consequence of their blackbox character is that we cannot use them to learn about the tasks they solve.

With respect to the originally proposed problem of *unsupervised phonological reconstruction*, not much has happened in the meantime. In 2013, Bouchard-Côté *et al.* (2013) showed that unsupervised automated phonological reconstruction is possible for Austronesian languages, using a complex framework in which stochastic transducers were applied to model the evolution of individual words across known reference trees. Since then, only Jäger (2019) has presented an alternative approach to the problem, in which methods for ancestral state reconstruction ( Nunn, 2011, 63–89) were applied to a test set of Romance languages. The results were apparently disappointing, but closer inspection easily shows that the problem is less the method itself (although a closer analysis would be needed), but even more the quality and the nature of the original data, which treats Latin as the ancestor of the Romance languages, although it has been known for a long time that a reconstruction of Romance languages cannot reveal Latin entirely, since many distinctions have been lost across all Romance languages ( Hall, 1950).

In summary, we can conclude that the problem of automated phonological reconstruction remains a difficult problem for which no satisfying solutions exist so far. Even the very good results reported for the supervised reconstruction on Romance languages should be taken with considerable care, since the original dataset by Ciobanu & Dinu (2014) is far too large to provide a realistic test case that would be applicable to other language families. Not only does it seem impossible to find a comparable number of cognate sets for other language families of the same time depth as that of the Romance language family, I would also suspect that the majority of supposed cognates in the data do not qualify as true cognates (referring to etymologically related words, see Trask, 2000, 63) but rather reflect late borrowings from Latin into the individual Romance languages. Since borrowings show very different rules of transformation, which are – at least in the case of borrowings from Latin into Romance languages – often much simpler than the complex sound change processes that can be observed in the languages of the world, it would be important to test the approach by Meloni *et al.* (2021) and the follow-up approach by Kim *et al.* (2023) on the much sparser dataset that we proposed for our shared task on reflex prediction in order to understand their real potential.

## Modeling problems

With respect to the modeling problems, there was – as far as I can judge – no substantial progress in the simulation of lexical change and sound change, but there were some interesting studies dealing with the problem of finding a statistical proof for language relatedness.

Ceolin (2019) tested the methods by Baxter & Manaster Ramer (2000), Kessler & Lehtonen (2006) and Kessler (2001) on a dataset consisting of six different language varieties, including Turkish, Mongolian, and Manchu, three languages that some scholars assume to be genetically related, showing that no conclusive results could be obtained in favor of the highly disputed Altaic language family ( Georg, 2017).

Kassian *et al.* (2021) employed the test reported by Turchin *et al.* (2010), originally inspired by Dolgopolsky (1964) to another dataste of languages from the disputed Altaic family, with the difference that they used reconstructed proto-languages. Their test also failed to provide conclusive evidence for the whole language family, although they argue that rather clear support can be found for a deeper relationship of the families tested by Ceolin (2019). While both Ceolin (2019) and Kassian *et al.* (2021) applied methods proposed before to newly compiled datasets, Blevins & Sproat (2021) designed a new workflow for to test for supposed deep language relations in order to find evidence for the hypothesis that the language isolate Basque is related to Indo-European. In contrast to previous studies, Blevins & Sproat (2021) test their approach on a rather large sample of languages where the language relations are known, showing that their approach is rather conservative, showing a tendency to reject grouping two languages within the same family in case of sparse evidence. Whether this is enough to prove the case of Basque and Indo-European, however, remains to be seen, since the data used in the test and the data used to test the potential relationship between Basque and Indo-European were not identical in design. This makes it more difficult to interpret the results of the test.

Given that Ceolin (2019) and Kassian *et al.* (2021) report contradicting results, both using methods that are supposed to deliver clear mathematical proofs, and given the difficulty to compare the evaluation study reported by Blevins & Sproat (2021) with the tests they conducted, I think it is safe to argue that the last word on the problem of finding a convincing statistical proof for language relatedness has not yet been spoken.

## Analysis problems

**Figure 2.**
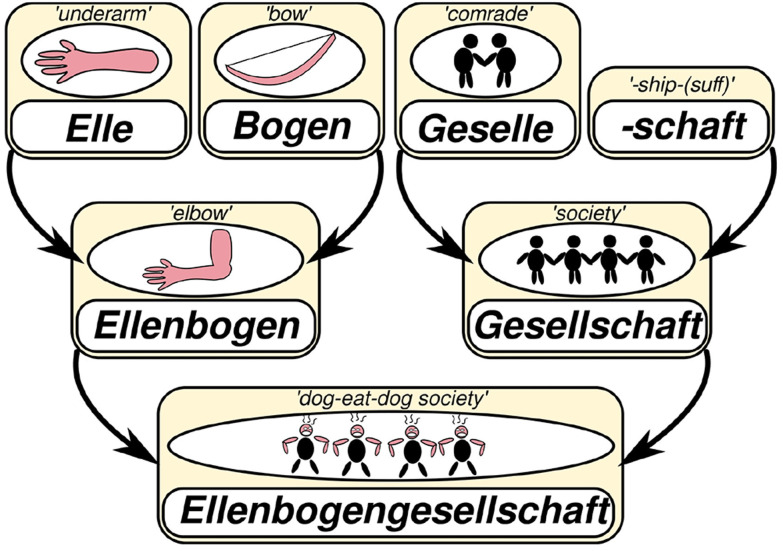
The lexical motivation of the term “dog-eat-dog society” in German.

No progress that I would be aware of has been made with respect to the establishment of first typologies for semantic change (Problem 8) and sound change (Problem 9). This confirms my original suspicion that both problems are indeed rather tough ones, requiring large amounts of annotated data – along with ideas of *how* the data should be annotated – that we simply do not have at the moment. While individual inquiries into semantic change based on corpus data of large well-documented language such as English have enjoyed a considerable popularity over the last years (e.g., Xu *et al.*, 2017), the problem I identified relates to cross-linguistic tendencies of semantic change, which cannot be observed by corpus studies for a few languages with a long documentation history. As a result, these corpus studies — as interesting as they may be — do not contribute to the solution of the problem I identified. Regarding the last problem on my list, the problem of establishing a typology of what I called *semantic promiscuity* by then, there has been no progress regarding the methodology of studying the phenomenon, but I think that there was at least some progress in explaining and defining the problem itself more properly. When I initially stated the problem, I was not aware of the rather large body of literature devoted to the topic of *lexical motivation*, referring to the process by which new words are created from existing ones ( Koch, 2001; Koch & Marzo, 2007; Urban, 2016). Most of the words in the lexicon of human languages are composed from other words and individual word histories can be very complex, as can be seen from the illustration in Figure 2, where I have described, how the term *Ellenbogengesellschaft* in German (“dog-eat-dog society”, lit. “elbow society”) derives from individual words and suffixes. For a long time, I have been trying to find a way to investigate to which degree the meaning of individual words contributes to their reuse.

Geisler (2018) makes the rather strong claim that word reuse results from bodily experiences made in early life, such as the act of “falling” or “standing”. This could explain why words build from the verbal roots meaning “to fall” and “to stand” are so frequently met in German. An alternative possibility would be that word reuse reflects actual trends that could change over time ( Alinei, 2001). This could explain, for example, the recent increase in the use of metaphors from psychology, popularly also known as “therapy speech” ( Prendergast, 2022). Most likely, none of the two extreme positions is absolutely true. Instead, it is quite likely that both actual trends and important (potentially bodily) experiences contribute to the reuse of words in the world’s languages. But a detailed investigation of the semantics underlying these processes by which new words are formed from existing ones has so far not been carried out yet.

When I first stated my problem of establishing a typology of semantic promiscuity, I did not know that quite some detailed work on the processes of *lexical motivation* – albeit mostly qualitative in nature – had already been carried out and that some authors had proposed concepts quite similar to what I had in mind when proposing the term *semantic promiscuity*. Blank (1997, 21), for example, describes the processes of *attraction* and *expansion*. Attraction refers to cases where a given concept “attracts” different words to express it. In theory, we might be able to measure the *attractivity* of concepts, that is, their propensity to be expressed by multiple words. Expansion refers to cases where a word receives new meanings. If one agrees that the *expansivity* of words typically depends on the meaning they express originally, one could take this idea one step further and measure the *expansivity* of concepts and compare it across languages. Taking it one additional step further, one could then ask not only which concepts are good at triggering the extension of a word’s meaning, but also which concepts are good at triggering the reuse of a word in word formation processes, which is what I meant to denote with the term “semantic promiscuity”.

In a recent study in which I proposed new methods for the automated inference of words that share certain parts resulting from word formation processes (called *partial colexifications* in that study, see List, 2023), I found that there is a tendency for words expressing certain concepts to be reused much more frequently in other words than words expressing different concepts. I also found a tendency for certain concepts to be expressed by words that are composed rather than being expressed by single morphemes. Since my analyses are based on a very rough approach that has not yet been tested any further so far, they should be taken with certain care. Given the confusion that the term “semantic promiscuity” has created in discussions with colleagues as well as in discussions following my original blog post ( List, 2019c), I decided – inspired by a comment of Alexandre François – to use the term “lexical root productivity” from now on, in order to refer to the reuse potential of words in the lexicon – resulting from their meaning.

Regarding the problem of establishing a typology of lexical root productivity, I would no longer consider this as the most important problem for the field of lexical typology. Instead, I think, that one could state the problem in broader terms as the problem of establishing a typology of processes of *lexical motivation* that would allow us to investigate both how words are reused across the languages in the world ( *form-based perspective*, also called *semasiological perspective*), and how concepts are expressed with the help of reusing lexical material ( *concept-based perspective*, also called *onomasiological perspective*). Despite earlier attempts to solve certain aspects of this problem ( Urban, 2012), a real typology of lexical motivation has not yet been established, and the problem can therefore be considered as an unsolved one.

## Outlook

In the end of 2018, I identified ten unsolved problems in computational historical linguistics that I considered as important and solvable at the same time. In my opinion, the importance of solving these problems has not changed since then. While quite some progress has been made in the past five years, most of the problems are still not solved, and it is not clear if and when we will find solutions for them. In order to be able to compare where I see the field of computational historical linguistics now, five years later, I have created a revised list of open problems, which is shown in Table 3. In two cases (Problem 3, contact layer detection, and Problem 10, typology of lexical motivation), I have decided to shift the focus and therefore modified the title of the problem. In two out of ten problems (Problem 3, automated borrowing detection, and Problem 4, automated phonological reconstruction), I would argue that some substantial progress has been made in the field, although I do not consider any of the problems as “solved” as of today.

**Table 3.** Updated list of open problems in computational historical linguistics.

No.	Problem (2019)	Problem (2023)	Progress
1	automated morpheme segmentation		-
2	automated sound law induction		-
3	automated borrowing detection	contact layer detection	+
4	automated phonological reconstruction		+
5	simulation of lexical change		-
6	simulation of sound change		-
7	statistical proof of language relatedness		(+)
8	typology of semantic change		-
9	typology of sound change		-
10	typology of semantic promiscuity	typology of lexical motivation	(+)

Given that my optimism from 2018, when I assumed that most problems could be solved in five to ten years’ time, has not turned out to be very reliable, I would now refrain from making any further assumptions on whether the ten problems outlined in Table 3 are solvable or not. If, however, in five years from now, the progress of the field of historical computational linguistics has been at a similar rate as it has been in the past five years, I assume that we will see some substantial progress, even if none of the problems can be solved completely.

## Data Availability

No data are associated with this study.

## Software availability

No software are required for this study.
