# Supplementary material for: Open Problems in Computational Historical Linguistics
Source: Open Res Eur. 2024 May 29;3:201. Originally published 2023 Nov 20. [Version 2] doi: 10.12688/openreseurope.16804.2 (PMC10864822; doi:10.12688/openreseurope.16804.2)
Supplement: Peer review history

# Reviewer report: Reviewer response for version 2

**DOI:** 10.21956/openreseurope.19255.r41040

**Published:** 2024-08-19

**Authors:** Michael Pleyer

**Affiliations:** 1 Center for Language Evolution Studies,, Nicolaus Copernicus University in Toruń, Toruń, Poland

The author has appropriately responded to my comments.  I accept that our views  on some of the issues surrounding language evolution and its perception differ, and the author presents a valid perspective even if I might disagree with some aspects. So I give my approval to the indexing of this article.

Is the argument persuasive and supported by appropriate evidence?

Yes

Is the work clearly and cogently presented?

Yes

Is the topic of the essay discussed accurately in the context of the current literature?

Yes

Does the essay contribute to the cultural, historical, social understanding of the field?

Yes

Reviewer Expertise:

Language Evolution, Evolutionary Linguistics, Cognitive Linguistics, Construction Grammar

I confirm that I have read this submission and believe that I have an appropriate level of expertise to confirm that it is of an acceptable scientific standard.

# Reviewer report: Reviewer response for version 2

**DOI:** 10.21956/openreseurope.19255.r41037

**Published:** 2024-06-07

**Authors:** Jeff Good

**Affiliations:** 1 University of Buffalo, Buffalo, USA

The revisions based on my feedback are appropriate in my view, and I appreciate the time the author took to respond to them.

Is the argument persuasive and supported by appropriate evidence?

Yes

Is the work clearly and cogently presented?

Yes

Is the topic of the essay discussed accurately in the context of the current literature?

Yes

Does the essay contribute to the cultural, historical, social understanding of the field?

Yes

Reviewer Expertise:

linguistics, historical linguistics, linguistic typology

I confirm that I have read this submission and believe that I have an appropriate level of expertise to confirm that it is of an acceptable scientific standard.

# Reviewer report: Reviewer response for version 1

**DOI:** 10.21956/openreseurope.18157.r37446

**Published:** 2024-02-22

**Authors:** Michael Pleyer

**Affiliations:** 1 Center for Language Evolution Studies,, Nicolaus Copernicus University in Toruń, Toruń, Poland

In this essay, the author discusses the current state of ten “open problems in computational historical linguistics” that he proposed in a series of blog posts in 2019 and envisioned as solvable within the next five years. Now, five years later he takes stock of the progress that has been made. He comes to the conclusion that although none of the problems have been solved, at least for some of them, progress has been made. This leads the author to slightly modify his list.

 This is an interesting essay, written in a personal style (which probably wouldn’t be appropriate for a research article but is fitting for an essay offering a personal perspective) that highlights a number of interesting open problems for computational historical linguistics, and historical linguistics in general. The author does a good job of relating these problems to the question of “big” and “small” problems in science and previous conceptualisations of the problems that historical linguistics should address.

 The two major issues I see relate to its discussion of language evolution and the field of language evolution. The author starts with a discussion of the question of language evolution and how it is seen by (historical) linguists. This discussion is interesting, but I felt that the connection to the next parts of the essay, the discussion of types of problems, and the ten specific open problems in computational linguistics could have been made clearer. What exactly does the discussion of language evolution contribute to the other two issues, “problems” and “problems in computational historical linguistics” and how are they related?

 The other major issue concerns the discussion of the state of language evolution research itself. It is certainly true, as the author attests from his own experience, that historical linguistics has mostly steered away from the question of the evolutionary emergence of language and the human language capacity. Here the author reports from his own experience, but the case might be further strengthened by also referring to published works that make this explicit, such as [1] introduction to historical linguistics:

 “Another topic not generally considered to be properly part of historical linguistics is the ultimate origin of human language and how it may have evolved from non-human primate call systems, gestures, or whatever, to have the properties we now associate with human languages in general. Many hypotheses abound, but it is very difficult to gain solid footing in this area. Historical linguistic theory and methods are very relevant for research here, and can provide checks and balances in this field where speculation often far exceeds substantive findings, but this is not a primary concern of historical linguistics itself.” (see also discussion in [4]).

 The author cites the 1866 “ban” by the Linguistic Society of Paris and states that “The situation has not changed since then.” This might indeed be true for the field of historical linguistics in general, but when taken to refer to linguistics as a whole this seems to me an overstatement. In general in language evolution research, the publication of [6] has been credited with initiating a broad revival of interest in the question, and by now language evolution research and evolutionary linguistics can be seen as strong interdisciplinary fields that also features many contributions by linguistics. There is a biannual “Conference on the Evolution of Language” (Evolang) and a *Journal of Language Evolution*, as well as an Oxford Handbook of Language Evolution [7] and a number of textbooks (e.g. [3]; [5]).

 In particular, the view of the impact of the ban stands in stark contrast to some published evaluations of it:

 “In the interim [before 1990], the story goes, all that happened was a comical series of silly unscientiﬁc hypotheses, nicknamed “bow-wow,” “heave-ho,” and “ding-dong” to expose their basic absurdity. *This view of the ﬁeld is a myth*. Darwin himself, and subsequent linguists such as Jespersen, made important contributions to this literature after the famous ban, and there was a major, important revival of interest in the 1960s and 1970s when many of the issues under discussion today were already debated insightfully (e.g. Hockett and Ascher, 1964; Hewes, 1973; Harnad et al., 1976).” [3].

 So again, I think with regard to historical linguistics, the author might be spot on, but with regard to the field of linguistics as a whole, I believe there have been significant changes regarding whether language evolution is a respectable area of inquiry, so I am not sure how true it is that as a whole “linguists would deliberately decide to ignore the investigation of a problem that some might consider the most fascinating the field has to offer.”

 The point that specifically for outsiders “ it is often very difficult to tell if a theory on the origin of language should be treated as a serious or a senseless idea” is very well-taken, but the essay’s juxtaposition of examples of this seemed a bit weird to me. It seems clear to me that Herder’s 1778 essay contribution on the origins of language should not be seen as a modern theory of language evolution, and that a theory invoking the concepts of *yin *and *yang* in a not very well-known journal, by authors who are not really active members of the language evolution community, should be treated with more skepticism than a careful consideration of the evidence for Neanderthal language capacities by highly-cited researchers.

 I am also a bit unhappy with the characterisation that there is “debate whether a single gene was responsible for our language faculty” as this is a minority view, and most discussions of FOXP2 revolve around its role in language evolution and the evolution of speech as one (important) contributing factor, and not the “language gene”, a view that has already been outdated in the 90s.

 Again, the point that there is a danger of bold and careless speculation is well-taken, but given the reality of the field of language evolution research as a whole, I think the framing of this point could be improved.

 It also seems to me just slightly too strong of a generalisation that “historical linguists never ask how many words a language has” – although saying that they “hardly ever ask” is probably correct. As one case in point, [2] makes reference to estimates of the vocabulary sizes of different societies. (The problem here of course is that such estimations such as the ones mentioned by [2] historically have been tied to racist and colonialist assumptions, which makes this issue a difficult one to navigate).

 But a short reference to this history of size assumptions in comparative linguistics (and its dangers) might be helpful for this essay in addition to the ones that are mentioned.

 There are also some smaller issues/minor comments, that can be easily addressed but should be addressed:

 - There are number of typos in the manuscript that should be taken care of, e.g.

 - p. 4 “loose” à “lose” p.6 “extend”, p.8 “was” à ways, p. 9 “workflow for to test for”, “dataset”

 - the plain language summary doesn’t make reference to the “computational” part of historical linguistics.

 - Page 4, fn 3, it might be good to mention that Dąbrowska (2020) is part of a bigger research programme of the author to investigate the question of “differences in competence” that she has also investigated for English, for example.

 - p.5/6 when discussing Hilbert problems, the author could make it a bit clearer if it is or can be related to the previous distinction of p- vs g-linguistics

 - Table 2: it might be useful to add to this table which of the problems are modelling, inference, and analysis problems (as is then discussed in the text).

 - p.10 In his discussion of the concept-based vs form-based perspective, the author might want to relate this to the traditional distinction in linguistics between a semasiological and an onomasiological perspective.

Is the argument persuasive and supported by appropriate evidence?

Yes

Is the work clearly and cogently presented?

Yes

Is the topic of the essay discussed accurately in the context of the current literature?

Yes

Does the essay contribute to the cultural, historical, social understanding of the field?

Yes

Reviewer Expertise:

Language Evolution, Evolutionary Linguistics, Cognitive Linguistics, Construction Grammar

I confirm that I have read this submission and believe that I have an appropriate level of expertise to confirm that it is of an acceptable scientific standard, however I have significant reservations, as outlined above.

## References

[1] : Historical Linguistics: An Introduction. *Edinburgh University Press* .1998; Reference source

[2] : Through The Language Glass: Why The World Looks Different in Different Languages. *Metropolitan Books Henry Holt and Company, LLC* .2010; Reference source

[3] : The Evolution of Language.2010; 10.1017/CBO9780511817779 10.1017/CBO9780511817779

[4] : Language change and language evolution: Cousins, siblings, twins?. *Glottotheory* .2020;11(1) : 10.1515/glot-2020-2003 10.1515/glot-2020-2003

[5] : Bibliography.2012; 10.1017/CBO9780511989391.011 267-301 10.1017/CBO9780511989391.011

[6] : Natural language and natural selection. *Behavioral and Brain Sciences* .1990;13(4) : 10.1017/S0140525X00081061 707-727 10.1017/S0140525X00081061

[7] The Oxford Handbook of Language Evolution.2011; 10.1093/oxfordhb/9780199541119.001.0001 10.1093/oxfordhb/9780199541119.001.0001

# Response

**Published:** 2024-02-22

**Authors:** Johann-Mattis List

**Affiliations:** University of Passau, Germany

Many thansk for this very inspiring response. I hope to find time soon to reply to all reports in a revised version of the essay. Here, I hope to be able to answer all points in a satisfying way.

# Response

**Published:** 2024-05-16

**Authors:** Johann-Mattis List

**Affiliations:** University of Passau, Germany

Many thanks for the critical remarks. I learned a lot from this review and found interesting new literature. The revised version addresses most of the points raised, I hope. Regarding the problem of coherency (why discuss language evolution and language origins at length, when then discussing concrete problems), I agree that this coherency may be lacking, I would however, feel reluctant to change the draft substantially in this regard, since — as other reviewers have also acknowledged — this is an essay, that should give me the possibility to jump in my lines of thoughts and to also report personal opinons and viewpoints that I would not present in this form in a proper research paper. I have added a reference to Campbell (1998) now, as an example for a text book introduction to historical linguistics that is very explicit on not treating language origin as a topic. I have seen that my statement on language evolution and language origin as scientific inquiries may have been a bit provocative and also not fair to the field. I have tried to modify the statements now. I hope that the demanded restriction to historical linguistics — including mainstream linguistics where historical linguistics is also usually ignored — makes the previous claims appear less general. The qualititative difference between the studies that I mention as examples is of course very obvious. But there are nuances one should not forget. While the yin-yang example is a strong outlier, one should place Herder into the historical context, and here, he was quite revolutionary, since he claimed that humans came up with the language faculty without a god. As silly as the theory may have sounded, in its pre-evolutionary context, the study was still quite important and freed the way for bolder demands. I have modified the passage now in such a way that I emphasize the difficulty of distinguishing serious from less serious attempts. The fact that some of the highly speculative proposals are published in big journals like Nature (think of the out of Africa discussion based on phoneme inventories) makes it in my opinion clear that a distinction is not that easy to draw between serious and non-serious research here. I have modified the wording on Fox-P and placed it into past tense, emphasizing that the debate is not recent. The fact that this research makes it into the big magazines like Cell shows again the suggestive force the topic bears, which is the main reason why it is discussed in this context. I hope that these modifications are sufficient to modify and adjust the potentially negative framing of the part on language evolution / origin in the previous version. Regarding the size of languages, I have modified the wording as suggested. Deutscher's book is an interesting reference for which I thank the reviewer. I have added a note mentioning it to the new version now. I have also addressed the problem of typos, and the minor points mentioned by the reviewer.

# Reviewer report: Reviewer response for version 1

**DOI:** 10.21956/openreseurope.18157.r36348

**Published:** 2024-02-15

**Authors:** John Nerbonne

**Affiliations:** 1 Germantistische Linguistik, Albert-Ludwigs Universität, Freiburg, Germany; 2 Computational Linguistics and chair of Humanities Computing, University of Groningen, Groningen, The Netherlands

Summary: Johannes-Mattis List (JML) provides an update on a paper from 2019 on ten outstanding problems in computational historical linguistics.  It will serve as an excellent survey for researchers in this field, and may help focus work there.

 Recommendation: Definitely index, drawing the author’s attention to (some of) the comments below, which he may care to take as reason to modify the paper a bit.

 General points: JLM refers several times to questions that will will/could/should be solved in the nearer (or farther) future. It would be interesting to hear him make the time references more concrete, e.g. in the next five vs. the next fifteen years.

 I liked the discussion of Weinreich et al.’s vs. Coseriu’s crucial questions, and they serve the sensible purpose of anticipating objections from readers.

 Issues:

 p.4, col.2 the size of a language. George Miller’s ‘The Science of Words” provides an estimate of the vocabulary size of Americans leaving high school, and he put it at 50K, many of which were derivationally related, using it to estimate how many new words were learned daily, and thence to why structure sensitivity was need to explain this.

 Comments & questions:

1. Abstract. Would it be sensible to identify the blog where these ideas were discussed?  This would be an indirect acknowledgment of contributions from blog participants.
2. pp. 3-4. I applaud the author’s attempt to identify significant problems in computational historical linguistics and thereby focus work on them. This is great. 

But I cannot resonate to an introduction that justifies this by claiming that the “driving force of all scientific inquiries are [sic] problems”.  I suspect that one could defend this against many objections by noting that attempts to find generality, for example, attack a “problem of too little generality”, but I also think, first– from an amateur’s view of the history of science-- that lots of advances have been stimulated by searches for generality or simplicity (a lot of physics in the last half century, chemistry in the Mendeleyev period), and second, that the focus on problem solving doesn’t characterize science alone. The Pragmatists Peirce and Dewey identified the solving of problems as the goal of all human thought.

 So, please keep the focus on the outstanding problems, but build up to it in a less highfalutin introduction.

 At the risk of repetition, this is a sensible and worthwhile paper.

1. p.7 “Inference problems. Maybe clarify that you’re aware that many morphologists reject the notion of morpheme, e.g., paradigm function morphology, and insist on the primacy of processes within paradigms. In the alternative view Umlaut (but also Ablaut) is a morphological process that isn’t comfortably reduced to the effect of a morpheme.
2. p.9 “Analysis problems” I was surprised that there was no mention of all the work in computational linguistics on semantic change. A collection on this, with many references: Tahmasebi *et al. *(2021 ^1^).

Form:  The paper is written clearly in nearly flawless English, which I appreciated.  Nonetheless, here are some details which might be improved.

- p.3,col.1 “working luckily” => working fortunately.
- p.3, col. 1 I’d prefer a translation of the Société’s ban on papers on the origin of language, “La Société n’adment aucune communication concernant, soit l’origine du langage, soit la création d’une langue universelle,”  as “the Société does not accept any contributions concerning either the origin.
- of the language faculty, or the creation of a universal”, i.e. instead of neither … nor
- p.4, col.1 “the amount of genes” => the number of genes   In general, ‘amount’ specifies quantity (of mass noun), ‘number’ quantifies plural count nouns.  But this is coming from an older, conservative writer.
- p.5, col ‘suggested to distinguish’ => suggested distinguishing.
- p.6, col.2, Table 2: Add to caption what I, m, and a indicate (Sci. Am.’s rules for captions: make graphics and table independently understandable, if possible.)
- p.6, Fig. 1 Explain significance of stars vs. squares vs circles, ditto blue vs. yellow stars.
- p.7, col.1, l.^3 closing parenthesis.
- p.7, col.2, Section “ New and …” last line: verb should be in present perfect.

Is the argument persuasive and supported by appropriate evidence?

Yes

Is the work clearly and cogently presented?

Yes

Is the topic of the essay discussed accurately in the context of the current literature?

Yes

Does the essay contribute to the cultural, historical, social understanding of the field?

Yes

Reviewer Expertise:

Computational Linguistics, Dialectology

I confirm that I have read this submission and believe that I have an appropriate level of expertise to confirm that it is of an acceptable scientific standard.

## References

[1] : Computational approaches to semantic change. *Berlin: Language Science Press.* .2021; Reference source

# Response

**Published:** 2024-02-15

**Authors:** Johann-Mattis List

**Affiliations:** University of Passau, Germany

Thanks a lot for this detailed and helpful review, I will try to make sure to answer to all points raised, once I sit down to work on the revision.

# Response

**Published:** 2024-05-16

**Authors:** Johann-Mattis List

**Affiliations:** University of Passau, Germany

This review has been very helpful to me and I hope that my modified version does justice to all remarks and points raise. I have tried to make clearer now when I think that problems may be solved (although I am hesitant to give concrete time frames). I was trying to identify the work by Miller but could not receive it in the original form for this revision. So I did not cite it in the current version, hoping also, that the reference to the more recent study by Nation (2021) is providing a suitable starting point for readers interested in the problem. The blog is linked now in the abstract. I have modified the characterisation of scientific inquiries as suggested by the reviewer. Problem solving is now one, but not the only scientific task. Inference problems have been clarified now. I have added another reference to a study by Xu et al (2017), that also illustrates how semantic change can be studied with corpora, emphasizing that these studies are particular language studies and not typological surveys. So one can do this research with English, but solving semantic change in English does not solve questions on semantic change in general. I have incorporated all hints on spelling errors and grammatical problems for which I am very grateful.

# Reviewer report: Reviewer response for version 1

**DOI:** 10.21956/openreseurope.18157.r37453

**Published:** 2024-02-13

**Authors:** Siva Kalyan

**Affiliations:** 1 Australian National University, Canberra, Australian Capital Territory, Australia; 2 The University of Queensland, Saint Lucia, Queensland, Australia

This essay starts by reflecting on the importance of open problems in setting research priorities in a scientific discipline, and points out the large difference between what non-linguists consider to be important questions about language (e.g. how “big” a language is) and what linguists consider to be important questions about language.

 The author then reviews preceding attempts to list the open problems in historical linguistics, and finds them all to be unsatisfying, as they do not translate into concrete research questions.

 The author then introduces his own list of ten open problems in computational historical linguistics, which he announced in January 2019. He reviews the progress that has been made on each of the ten problems since then, and concludes while there has been substantial progress towards solving two of the problems (automated borrowing detection and automated phonological reconstruction), the problems are far from being solved. He also revises the formulation of two of the problems, to better reflect his understanding of the field.

 Overall, I find the first three sections a bit lengthy, and would prefer the author’s list of open problems to be introduced sooner. However, if the audience for this essay includes non-linguists (or non-historical linguists), then the first three sections contain valuable background information.

 My only suggestion for improvement is that the replacement of the term “semantic promiscuity” with “lexical root productivity” should be introduced earlier, since the former term is very confusing, and especially so for non-linguists.

Is the argument persuasive and supported by appropriate evidence?

Yes

Is the work clearly and cogently presented?

Yes

Is the topic of the essay discussed accurately in the context of the current literature?

Yes

Does the essay contribute to the cultural, historical, social understanding of the field?

Yes

Reviewer Expertise:

Computational historical linguistics

I confirm that I have read this submission and believe that I have an appropriate level of expertise to confirm that it is of an acceptable scientific standard.

# Response

**Published:** 2024-02-15

**Authors:** Johann-Mattis List

**Affiliations:** University of Passau, Germany

Many thanks for these insightful comments, which I'll try to work into a revision of the essay.

# Response

**Published:** 2024-05-16

**Authors:** Johann-Mattis List

**Affiliations:** University of Passau, Germany

Again, many thanks for this very positive review. In the revised version, I have tried to address the point of being more explicit -- earlier in the text -- about lexical root productivity.

# Reviewer report: Reviewer response for version 1

**DOI:** 10.21956/openreseurope.18157.r37450

**Published:** 2024-01-31

**Authors:** Thomas Brochhagen

**Affiliations:** 1 Universitat Pompeu Fabra, Barcelona, Catalonia, Spain

This work discusses motivations and progress made toward addressing ten problems in historical computational linguistics, originally proposed by the author five years ago. I see the contribution as twofold. On the one hand, as argued within, there is use in taking a step back and evaluating where the field stands; what challenges it faces; and how they may be solved. On the other, after establishing a set of challenges, there is worth in iteratively looking back and evaluating to which extent these challenges have been solved; adjusting the problems and their framing as necessary.

 Overall, I believe the manuscript in its present form already convincingly achieves the above so I'll just raise a few minor issues for the consideration:

 1. I believe the Introduction is too pessimistic. First, while the ban from the Societe de Linguistique de Paris may have had its impact and is striking in its own right, it was abandoned within a decade and is best judged in the historical context it happened in (see, e.g., https://doi.org/10.1142/9789814401500_0133 for pointers). Second, and more importantly, much interesting and productive research on the evolution of language (both in terms of its emerge and in terms of the dynamics of change involved) has been conducted in the last decades. It may be that many proposals/models will turn out to be wrong in one way or another but, as I see it, that's the currency of science. The tone of the manuscript (particularly around the 4th paragraph) struck me as dismissive and as a bit of caricature. We can agree that there is outrageous speculation in the field, but I don't think this area of research is unique in this respect. I'd encourage the author to elaborate more on their stance, if it is indeed as pessimistic as it sounds in the current version, or to consider attenuating this part a little.

 2. There is little to no discussion on "automated morpheme segmentation". I found this curious considering that (i) subword tokenization is an important component of the NLP-pipeline of all successful Large Language Models, which are also increasingly becoming multilingual (see, e.g., mT5 [ https://github.com/google-research/multilingual-t5] or BigScience's Bloom [ https://bigscience.huggingface.co/blog/bloom]); and that (ii) since 2019 morpheme segmentation has, for instance, figured as a shared task in the Special Interest Group on Computational Morphology and Phonology ( https://sigmorphon.github.io/sharedtasks/). It may be that these NLP-oriented approaches are not what the author had in mind since they are often not linguistically motivated. But they do fit the manuscript's preference for unsupervised solutions. I was left wondering what they lack to not even be considered solutions toward the challenge.

 3. I concur with the Jeff Good ( https://doi.org/10.21956/openreseurope.18157.r36345) that (i) the manuscript would benefit from careful proofreading to eliminate mistakes (e.g., "[...] we may easily loose the chance us to enrich our [...]" on p. 4) and that, in particular, (ii) Figure 2 would benefit from some further context to be interpreted as intended, either in the caption or in the text.

Is the argument persuasive and supported by appropriate evidence?

Yes

Is the work clearly and cogently presented?

Yes

Is the topic of the essay discussed accurately in the context of the current literature?

Yes

Does the essay contribute to the cultural, historical, social understanding of the field?

Yes

Reviewer Expertise:

computational cognitive modelling; Bayesian methods; lexical typology

I confirm that I have read this submission and believe that I have an appropriate level of expertise to confirm that it is of an acceptable scientific standard.

# Response

**Published:** 2024-02-15

**Authors:** Johann-Mattis List

**Affiliations:** University of Passau, Germany

Thanks a lot for these very helpful comments which I'll try to make sure to include in a revision of the essay that I am planning to submit soon.

# Response

**Published:** 2024-05-16

**Authors:** Johann-Mattis List

**Affiliations:** University of Passau, Germany

Thanks again for this very useful review. I have now finished my revision and tried to address the points raised. Specifically, regarding the point that I was too pessimistic about language evolution, I have tried to modify the wording now. I agree that my introduction was pessimistic, and I have modified this statement now, by also emphasizing that it is my personal opinion and that it reflects the impression I have from the research situation in Germany, but I think that given the essay character, it is justified to share this impression. I think my harsh judgment on the field is also particularly justified since linguistics has not managed to bridge the gap of several hundred thousand years since language evolved the first time and where out classical methods for historical language comparison date back. While we have a lot of convergence regarding the theories of particular language families, we do not find a communis opinio on how language originated. We do not even find a concrete scenario in the current research devoted to the topic. Regarding the topic if subword tokenization and morpheme segmentation, I hope the new version addresses this problem appropriately. The problem of subword tokenization is that it does not solve the problem at hand. We are talking about a very specific problem here, where the goal is to find the morphemes in a list of about 1000 words of a given language. In the original blog post I reference, it is illustrated (and this has not changed until now) that tools like Morfessor fail to identify morphemes when given only 1000 words of a German text. Subword tokenization also does not address this task, since the goal consists in a concrete solution of the problem, not in finding the most frequent forms in a text. I have clarified this in the revised text. Errors in the form of typos have hopefully also all been accounted for now.

# Reviewer report: Reviewer response for version 1

**DOI:** 10.21956/openreseurope.18157.r36345

**Published:** 2024-01-08

**Authors:** Jeff Good

**Affiliations:** 1 University of Buffalo, Buffalo, USA

The core of this paper provides an update on progress towards a solution to ten problems in computational historical linguistics that were proposed by the author in 2018/2019. It additionally provides more general discussion of the role of problems in advancing scientific research following the tradition of Hilbert's problems in mathematics, and much of the framing of the contribution of the paper is in terms of "big picture" questions about problems in science even if the problems outlined for computational historical linguistics in the paper are somewhat narrower in scope. The paper is somewhat unusual in the context of linguistics due to personal nature of the rhetorical framing. However, this seems reasonable since it is being submitted under the category of "essay".

 Because the author has already made substantial contributions to computational historical linguistics, I believe that it would be good to see this essay accepted in some form since his ideas about major problems in this area should be of general interest, though I would recommend certain changes. Most of these represent relatively minor issues centering around clarification of the manuscript, but two are more significant. I'll discuss those first.

 More significant issues:

 1. As discussed above, the focus of the paper is the ten problems in computational historical linguistics that were proposed by the author in 2018/2019. While a revised set of problems is presented here, this set is still more or less based on the original set. I think, in a paper like this, it would be beneficial for the author to also consider whether he would still propose the same basic ten problems proposed in 2018/2019 as being the most significant problems today. Perhaps, for example, some of them seem less important now than they did before, and there are only (say) eight "key" problems in his view. Or, perhaps, there is an eleventh problem he thinks should be added.

 2. I think that, for the arguments of the paper to be clearly interpretable, it would be important for there to be a clear definition of what the author believes "computational historical linguistics" is, what its key goals are, and what model it assumes for "language" and "language change". In practice, for example, my sense is that most work in computational historical linguistics has, as its key goal, linguistic reconstruction, and this is suggested by work such as Jaeger (2019). Achieving this goal also means arriving at a better understanding of language change, but that does not usually seem to be the main goal of such work but, rather, a means of achieving the goal of reconstruction. Put differently, computational historical linguistics seems to emphasize the reconstruction of prehistory more than, for example, developing universal theories of sound change. I have no particular idea regarding what "computational historical linguistics" should include (or not include), but I do think it would be good for a paper like this one to take a clear position on this.

 Minor issues:

 - I noted various minor typos in the manuscript. Before it is accepted, it should be proofread carefully **.**

 - "Non-linguists are often very surprised that asking for the origin of language is a taboo question in the field of historical linguistics." I don't have the impression that this is a taboo anymore. Rather, my sense is that it is understood to be outside of the domain of much of historical linguistics which is focused on how languages changed after language (as we understand it) had developed. This is, in large part because that's what the methods of historical linguistics are designed to investigate. The evolution of language is now studied, of course, but it tends to be done outside of historical linguistics in a narrow sense.

 -The summary given of Weinreich et al.'s (1968, 183–187) problems does not fully match my reading of that work. For example, I don't read the "evaluation problem" as involving the degree to which change happens "consciously" but, rather, as whether language communities are aware that a change is taking place (even if it is originating in an unconscious way). I think it would be good to double check this summary. Similarly, I have always interpreted the "actuation problem" as being a version of "why do languages change". Maybe the author is correct in saying that this is not the case, but double checking the interpretation would still be a good idea in my view.

 -"The failure to distinguish questions pertaining to particular languages and questions pertaining to language in general has led to many misunderstandings in the field of comparative linguistics." Can some examples be cited here?

 -Table 2: I don't understand how automated morpheme segmentation is a historical computational problem rather than a general linguistic one. Can that be clarified?

 -Figure 1: Can the interpretation of 20 x, 10 x, and 5x be explicitly explained in the text?

 -"As an example for this procedure, consider the problem of cognate detection, the detection of historically related – or homologous – words across languages." This paper seems to be aimed at a general audience, not linguistic specialists. If so, I think the description of the concept of cognate needs to be expanded since it is discipline specific.

 -"Based on this evolutionary model, we can then conclude that similar words observed across different language varieties have been inherited from the common, formerly unified, ancestral variety." Given that the paper opens with a discussion of the question of the evolution of language (from non-language), it might be best to avoid the term "evolutionary model" here since "evolution" has an ambiguous meaning in historical linguistics.

 -"...but it requires a model of language relatedness that can then be tested against a random model in which languages are thought to be unrelated.": What is meant by "language relatedness"? It seems like what is meant is "genealogical relatedness under so-called normal transmission" (as opposed to, say, sharing features through contact). I think it would be good to make that explicit.

 -"While there are numerous attempts in the literature to come up with a convincing statistical model to prove genetic relationship..." (footnote 5): I would add Nichols (1996) to this list.

 -"Rather huge progress – at least when looking back specifically at the last couple of years – has been made with respect to supervised phonological reconstruction.": I think it would be useful to say something about the supervision involved in these approaches.

 -"While scholars had been working on this problem before, a first impressive demonstration of the capability of machine learning methods...": I think that it is worth mentioning that these are of a different character than other approaches since there is the so-called "black box" effect with them. That is, they may work well, but we don't really know why. This is opposed to computational models that require an amount of computation that would be practically impossible for humans to conduct by hand, but where each step in the process is known and understood.

 -"I identified ten unsolved problems in computational historical linguistics that I considered as important and solvable at the same time." Following on from my general comment above, would the author still consider all of these important (or as important) today?

Is the argument persuasive and supported by appropriate evidence?

Yes

Is the work clearly and cogently presented?

Yes

Is the topic of the essay discussed accurately in the context of the current literature?

Yes

Does the essay contribute to the cultural, historical, social understanding of the field?

Yes

Reviewer Expertise:

linguistics, historical linguistics, linguistic typology

I confirm that I have read this submission and believe that I have an appropriate level of expertise to confirm that it is of an acceptable scientific standard, however I have significant reservations, as outlined above.

## References

[1] : Computational historical linguistics. *Theoretical Linguistics* .2019;45(3-4) : 10.1515/tl-2019-0011 151-182 10.1515/tl-2019-0011

[2] : The Comparative Method Reviewed : Regularity and Irregularity in Language Change. *oxford university press* .1996;

# Response

**Published:** 2024-02-15

**Authors:** Johann-Mattis List

**Affiliations:** University of Passau, Germany

Thanks a lot, these comments are very helpful, and I will try to make sure to reply to all of them when doing my revision.

# Response

**Published:** 2024-05-16

**Authors:** Johann-Mattis List

**Affiliations:** University of Passau, Germany

Thanks a lot for this very useful review of the essay. In my revision, I have tried to address all points mentioned. I summarize major modifications below.

1. I have added one sentence clarifying that due to the fact that the problems have not been solved, I would not revise the list by now.
2. I have added a sentence explaining what I understand when using the term "computational historical linguistics". My definition in this context is much more narrow than trying to find a computational solution to questions on sound change, but rather aims to cover those approaches that try to formalize with a computer what people have been doing manually so far.
3. I have tried to account for all errors and hope I have identified most of the ones that were noted by the reviewer.
4. Regarding the origin of language, I have added a statement now, also since other reviewers were not happy with my wording, where I emphasize that it is my personal experience that the question is avoided, and also emphasized that it may be restricted to the context of Germany, where we have a tradition of distinguishing evolution and language origins from language history and language development in terms of the terminology we use.
5. Regarding Weinreich et al.'s article, I am a afraid that I do not know how to read the "why" in "why languages change". There are two answers here, following the notion by Coseriu, namely "for what goal" (why is it useful to change) or "what specific aspect of the nature of languages triggers their change, of which we observe that it always happens?". In this context, I think that my reading of the "actuation" problem as referring to the triggers of change seems to hold to me, but if it can be further specified where my reading fails, I would be very glad and change it accordingly. Regarding the evaluation problem, my intention was to specifically point to the reading the reviewer suggests, so I changed that part in the text.
6. I have tried to address the problem of distinguishing p- and g-linguistics. I have made an attempt to specify by pointing to methods that would only work on one orthography. Examples with references are not mentioned here, since the majority of encounters is in review situations. One could extend the passage by referring to the problem of equating English with Language, but I hope it is clearer already in the current form.
7. I have clarified why morpheme segmentation is important in historical linguistics.
8. I have added a concrete example to the example on cognates (German and English).
9. I avoid the term "evolutionary model".
10. I specify what I mean by language relatedness ("common ancestry").
11. Regarding Nichols (1996): The study could be added, but since the work is usually not cited in this context, since the theory proposed in this paper cannot be quantified (it is an example, but nobody has made a computer model out of it, unlike the work by Baxter and others mentioned in this context), I'd prefer to not quote the article in this context.
12. Supervised vs. unsupervised approaches are now explained (I hope it is in sufficient detail).
13. A short statement on the black box character of some appraoches has been added.
14. I try to be more explicit regarding the importance of the problems in my work and if I see new ones by now.
